# Characterization of CD46 and β1 integrin dynamics during sperm acrosome reaction

**DOI:** 10.1038/srep33714

**Published:** 2016-09-26

**Authors:** Michaela Frolikova, Natasa Sebkova, Lukas Ded, Katerina Dvorakova-Hortova

**Affiliations:** 1 Group of Reproductive Biology, Institute of Biotechnology CAS, v.v.i., BIOCEV, Prumyslova 595, 252 50, Vestec, Czech Republic; 2 Department of Zoology, Faculty of Science, Charles University, Vinicna 7, Prague 2, 128 44, Czech Republic; 3 Department of Cell Biology, Faculty of Science, Charles University, Vinicna 7, Prague 2, 128 44, Czech Republic

## Abstract

The acrosome reaction (AR) is a process of membrane fusion and lytic enzyme release, which enables sperm to penetrate the egg surroundings. It is widely recognized that specific sperm proteins form an active network prior to fertilization, and their dynamic relocation is crucial for the sperm-egg fusion. The unique presence of the membrane cofactor protein CD46 in the sperm acrosomal membrane was shown, however, its behaviour and connection with other sperm proteins has not been explored further. Using super resolution microscopy, we demonstrated a dynamic CD46 reorganisation over the sperm head during the AR, and its interaction with transmembrane protein integrins, which was confirmed by proximity ligation assay. Furthermore, we propose their joint involvement in actin network rearrangement. Moreover, CD46 and β1 integrins with subunit α3, but not α6, are localized into the apical acrosome and are expected to be involved in signal transduction pathways directing the acrosome stability and essential protein network rearrangements prior to gamete fusion.

A great deal is known about the mammalian fertilization, and all the physiological changes that male gamete must undergo in order to be able to fertilize the egg. However, the actual sperm protein dynamics that precedes the interaction with the egg, is still covered in a veil of mystery. So far, many proteins have been selected to be the sperm-egg binding and/or fusing candidates, some of them (Izumo1, CD9, Juno) were proven to be essential, some of them were discovered to play an unsuspected new role (CD46, tetraspanins)^1^. These new proteins are predicted to be also involved in the actual membranes’ fusion, covering not only reproduction but also vesicular trafficking, immune reaction and neurotransmission. There are several crucial physiological checkpoints before the sperm fuses with the egg. Determining molecular mechanisms important for sperm-egg membrane interaction is the major challenge to current reproductive biology, with significant importance to human assisted reproduction. Furthermore, it would also be of interest for the field of neuro-physiology, immunology, cell biology and even cancer research, by stretching the understanding of the membrane fusion process in general, beyond the most well understood virus-cell and intracellular vesicle fusion^2^.

This study focuses on the final, but the most dramatic mature sperm metamorphosis, called the acrosome reaction (AR), which is characterized by the controlled exocytosis of the single giant enzyme-rich secretory vesicle the acrosome. This is a critical Ca^2+^-dependent event that follows capacitation, enabling sperm to be fusion-competent. During this event, the plasma membrane of the acrosomal area of the sperm head fuses with the outer acrosomal membrane, intra-acrosomal proteins are released into the extracellular space and new protein domains appear on the surface of the sperm head^3^,^4^. The formation of the lipid raft clusters in the plasma membrane, during sperm maturation called capacitation, present a preferential site of hybrid vesicle formation during acrosomal secretion. This structural organization resembles the secretory vesicles of neurons and somatic cells^5^. Accordingly, acrosomal secretion shows remarkable parallels to the active zone of the presynaptic terminal in neurons where neurotransmitter vesicle fusion occurs and thus may be termed “acrosomal synapse”^6^. Many of the proteins present in the plasma membrane and in the outer acrosomal membrane lipid rafts are re-localized or lost^3^,^7^,^8^,^9^, along with dramatic changes in the organization of the cytoskeleton, which are an equally important part in this event^10^,^11^.

Although a function of cytoskeletal proteins, mainly actin, is well known during the AR, it remains unclear which proteins participate in directing the dynamics of its organisation. CD46 (membrane cofactor protein, MCP) is expressed broadly on the surface of somatic cells in humans and plays a pivotal role in the cell’s self-protection against the complement, which is also used by tumour cells^12^. However in sperm, CD46 is present on the acrosomal membranes and it is not surface exposed until the AR is completed, which suggests a new potential role of this protein. CD46^−/−^ deficient males show a higher rate of spontaneous acrosome reaction compared to wild type males^13^. This finding led to the theory that CD46, via actin, may play a role in the stabilization of the acrosomal membrane^13^,^14^,^15^ and consequently the whole acrosome region. The cytoplasmic domain of CD46 contains several phosphorylation and signalling motives^16^ and this protein is known to play an important part in the signalling pathway in different types of somatic cells^17^,^18^,^19^. It was also proved that CD46 could induce large cytoskeleton reorganization in epithelial cells and the T-cell^20^. It may imply that CD46 could also affect actin arrangement in sperm during the AR and play an active part in the rearrangement process, as it has been shown in somatic cells, where actin reorganization is affected either through specific protein kinases by CD46^17^,^21^ or via its binding partners such as β1 integrin subunit^22^,^23^,^24^. Integrins are transmembrane proteins consisting of α and β subunits and they play an active part in signal transduction pathways and mediate specific cell-cell/cell-extracellular matrix interactions. Integrins are also present in sperm^25^ and as well as CD46 they possess the ability to influence the actin reassembly. These heterodimers serve as membrane receptors, which mediate the signal, both into and out of the cell^26^. β subunits of integrins directly or indirectly bind to actin and therefore they play a key role in controlling actin remodelling^26^,^27^. It is known that integrins are often associated with other membrane receptors in multi-molecular complexes that participate in cell activation^28^,^29^,^30^.

One of these multi-molecular complexes, where integrins participate, was detected on the egg plasma membrane and it is called – the tetraspanin web. The tetraspanin web is a complicated protein network formed by the interaction of members of the tetraspanin family and others proteins^31^,^32^,^33^. It is known that integrins, such as α6β1, form clusters on the egg plasma membrane at the site of sperm contact^34^ and they are significantly relocalized after fertilization^35^ in a similar way as sperm β1 integrins relocate into the area of fusion during the AR^25^. Interaction between egg α6β1 integrins and egg tetraspanins, mainly CD9 (an egg key player in gamete interaction)^36^,^37^, was reported in the oolema^38^. Besides binding to integrins, CD9 is also able to bind to CD46^22^,^23^.

The interaction of CD46 and β1 integrins is well known in somatic cells, including humans^22^, but there is no information about their interaction in sperm, however, due to the complexity of both proteins, their involvement in gamete attachment could be suspected. Hereby, we would like to present that in mouse sperm, CD46 and β1 integrins are binding partners and could play a crucial role in directing the onset of the acrosome reaction via their interaction with the actin cytoskeleton. Also, we suggest that the tetraspanin web or very similar structures could exist on sperm, and CD46 and integrins are part of them.

## Results

### CD46 and β1 integrin relocation

To identify the behaviour of the studied proteins, CD46 and β1 subunit of integrins, during sperm maturation and acrosome reaction, we firstly examined immunohistochemicaly their localization in the sperm head in freshly released epididymal sperm. We were interested whether CD46 would be confined only to the acrosome region of the sperm head, as previously reported^15^, or would it display a tetraspanin-partner like dynamic behaviour over the time of capacitation and/or AR. Similarly, we were interested in the pattern of β1 integrin and aimed to compare the nature of both proteins under *in vitro* defined conditions.

**Figure 1:**
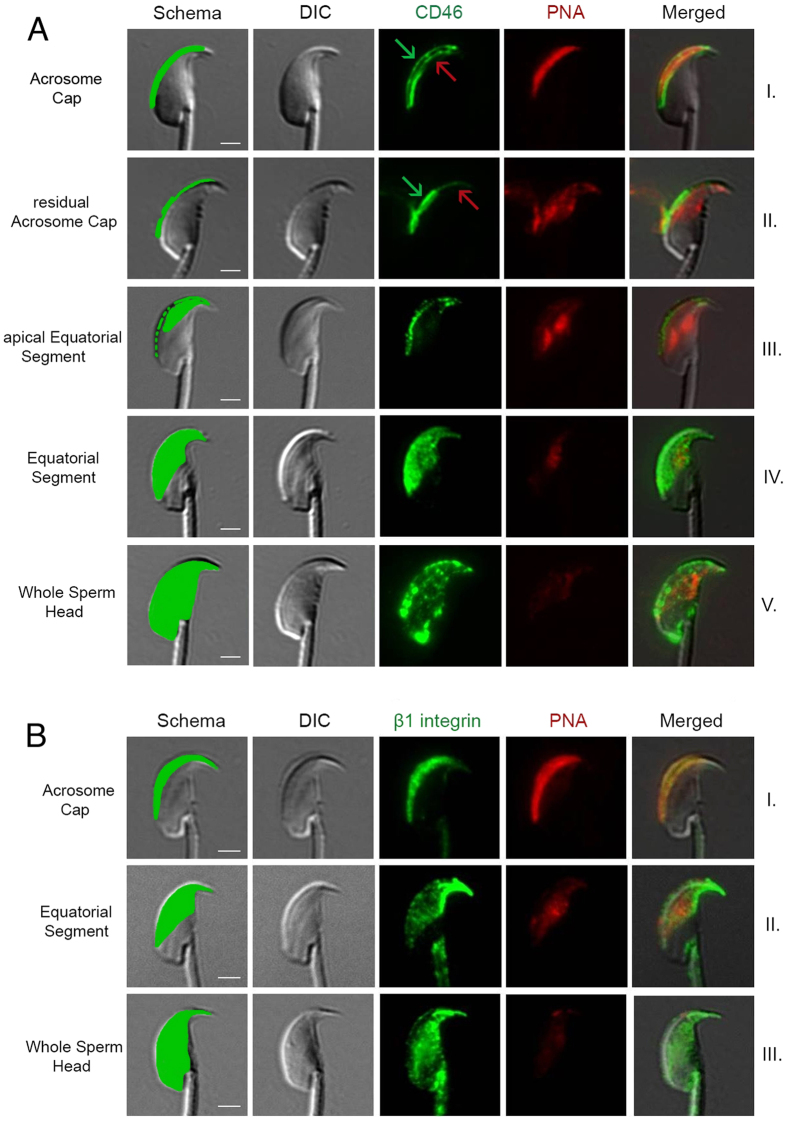
Progress of CD46 and β1 integrin relocation prior and during AR. (**A**) CD46 (green), PNA lectin (red). (**B**) β1 integrin (green), PNA lectin (red). The first column represents a schematic illustration of (**A**) CD46, (**B**) β1 integrin localization in the acrosome intact sperm (line I) and in sperm during the AR progress. (**A**) CD46 detection in intact acrosomal membranes (line I), and the residual outer acrosomal membrane (line II) (see the green arrow), the inner acrosomal membrane begins to emerge (see the red arrow). CD46 relocation progress during the AR is seen across the apical equatorial segment towards the whole equatorial segment and the whole sperm head. (**B**) β1 integrin is relocated across the apical equatorial segment towards the whole equatorial segment and the whole sperm head. In contrast to CD46, the residual acrosome cap and the apical equatorial segments were not detected. Scale bar represents 2 μm.

**Figure 2:**
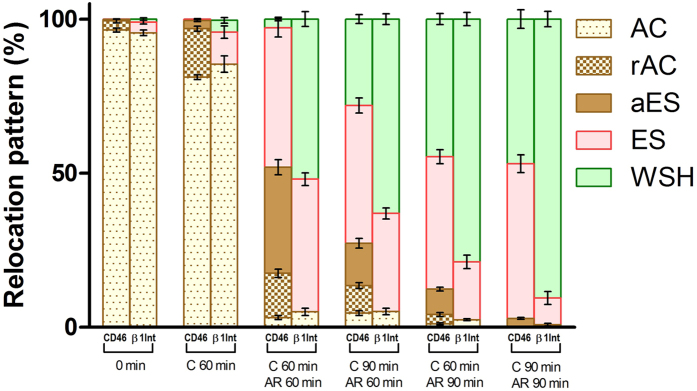
Percentage distribution of individual CD46 and β1 integrin relocation patterns among different times of capacitation and induced AR. Individual bars denote the percentage distribution of CD46 and β1 integrin staining patterns among individual times of capacitation and induced AR. Error bars denote SEM. AC – Acrosome Cap, rAC – residual Acrosome Cap, aES – apical Equatorial Segment, ES – Equatorial Segment, WSH – Whole Sperm Head. C – time of the capacitation, AR – time of the induced acrosome reaction.

Specific monoclonal antibodies (mAbs), see methods, were used to label the individual proteins and follow their localization over the sperm head. Epifluorescent microscopic observation detected distinct sperm head regions that showed a profound distribution of both proteins (Fig. 1) during the acrosome reaction, but not during the capacitation. However, the protein relocation depended on the length of capacitation, whether 60 or 90 minutes. When sperm were left to capacitate for 90 min, the protein relocation during acrosome reaction was faster compared to the group capacitated for only 60 min (Fig. 2). CD46 displayed five clearly distinct labelled regions, such as the acrosome cap, residual acrosome cap, apical equatorial segment, equatorial segment and the whole sperm head (Fig. 1A). For the β1 integrins, only three patterns could be followed, such as the acrosome cap, equatorial segment and whole sperm head (Fig. 1B, S1a,b).

The CD46 and β1 integrin localization during the 60 or 90 min AR progress was clearly similar. Prior to the AR, or at its very beginning, both proteins could be detected in the apical acrosome cap (Fig. 1A,B line I) with a visible membrane location (Fig. 1A arrows). As the AR progressed, the other regions became positive for both proteins, as shown in Fig. 1A,B lines II-V for CD46 and II-III for β1 integrin). The acrosome PNA labelling was only residual or absent when proteins entered the equatorial segment, and always absent when the whole sperm head was positively labelled. This could be interpreted that the studied protein relocation is triggered by the onset of the AR, but carries on after the acrosome content release.

There was also about 15% of acrosome reacted sperm during 60 min capacitation *in vitro*, which is in correlation with previous findings^14^. In this sperm fraction, the beginning of the protein relocation was detectable, and progressed as far as the apical equatorial segment in the case of CD46 or even over the whole sperm head for β1 integrins.

**Figure 3:**
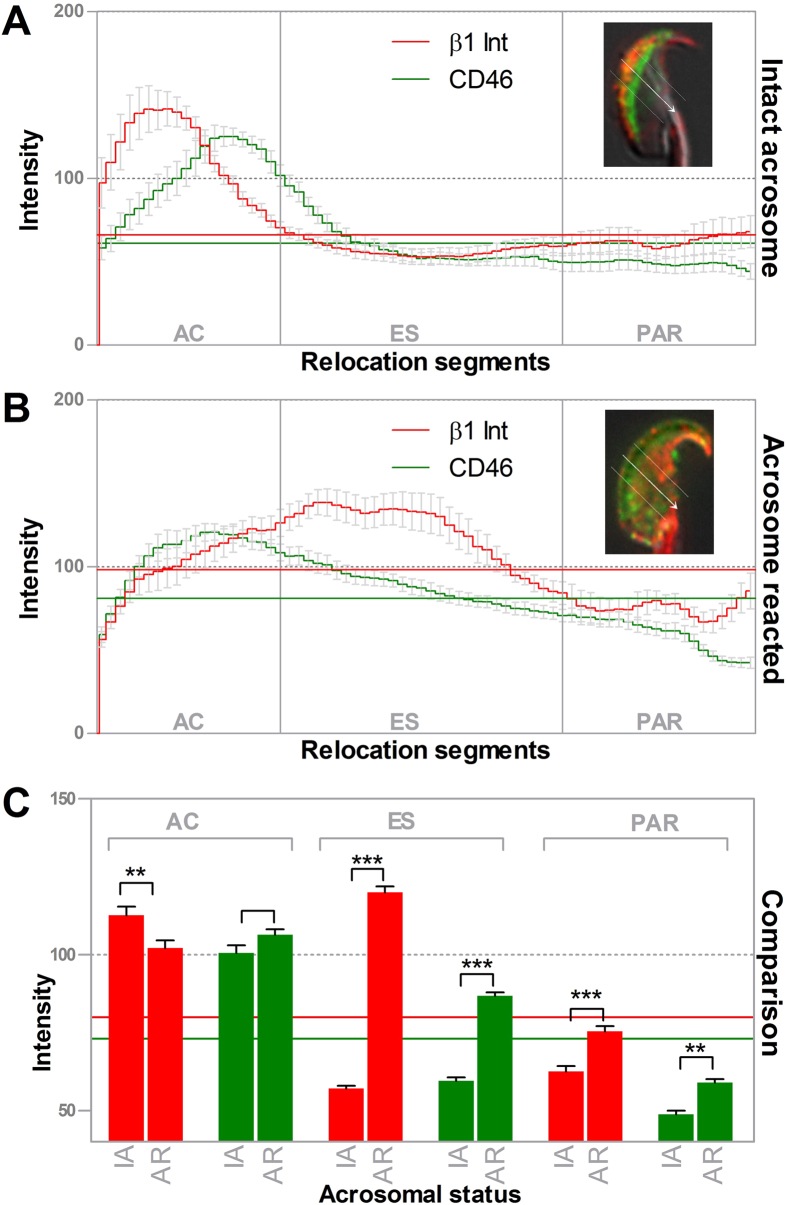
Statistical analysis of the relocation process. (**A**) Coloured lines with error bars represent the relative fluorescent intensities of β1 integrin (red) and CD46 (green) among the individual segments of the sperm head in 10 sperm with an intact acrosome. Horizontal coloured lines represent the arithmetic means of the fluorescent intensities for β1 integrin (red) and CD46 (green). (**B**) Coloured lines with error bars represent the relative fluorescent intensities of β1 integrin (red) and CD46 (green) among the individual segments of the sperm head in 10 acrosome reacted sperm. (**C**) Statistical comparison of the relative fluorescent intensities of β1 integrin (red) and CD46 (green) among individual segments of the sperm head between the acrosome-intact and acrosome-reacted sperm. Horizontal coloured lines represent the arithmetic means of the fluorescent intensities for β1 integrin (red) and CD46 (green). Error bars denote SEM. AC – Acrosome Cap, ES – Equatorial Segment, PAR – Post-Acrosomal Region. p value equal or lower than 0.05 was considered to be significant, *p ≤ 0.05, **p ≤ 0.01, ***p ≤ 0.001.

To analyse the distribution of individual CD46 and β1 integrin relocation patterns, among groups with different times of capacitation followed by the induced AR, the depicted protein patterns were placed beside each other (Fig. 2). The proteins behaviour was shown to be similar. This was further confirmed by the statistical analysis of fluorescent intensities, which depicted fluorescent signal differences between protein distribution in intact acrosome and in acrosome reacted sperm (Fig. 3). Both analyses are described in the following chapter.

### Quantitative analysis of the relocation process

The graphical and statistical output of our data is presented in Figs 2 and 3. In detail, Fig. 2 is represented by a column chart, where individual columns display the percentage distribution of the individual CD46 and β1 integrin relocation patterns among different times of capacitation and induced AR. At the onset of *in vitro* capacitation, the majority of sperm display the acrosome cap (AC) pattern for both CD46 and β1 integrin, which reflects the localisation of appropriate molecules in an intact acrosomal membrane. During the first 60 min of capacitation, the majority of sperm still kept the AC pattern, but the percentage of patterns related to the relocation of β1 integrins to the equatorial segment, and CD46 to the residual acrosome cap (rAC) and the apical equatorial segment (aES) slightly increased, which was expected, due to a well-known spontaneous AR in mice that represents over 10% of the sperm population. The following induction of AR by calcium ionophore caused a dramatic change in the relocation dynamics for both CD46 and β1 integrin molecules. Only less than 10% of sperm now displayed the AC pattern, and on the other hand, the pattern related to the relocation of a fluorescent signal to the equatorial segment (ES) was most prominent for the CD46 molecule. β1 integrin showed even more rapid relocation dynamics with a positive pattern detectable over the whole sperm head (WSH), including the post-acrosome region (PAR) as the prominent one. In subsequent times of analysis, the percentage of sperm expressing relocation of CD46 and β1 integrins into ES and post-acrosome region covering the whole sperm head continually increased. β1 integrins still carried on with a slightly more time dependent rapid progress of its relocation to ES and PAR compared to CD46.

To further support our findings about protein relocation dynamics obtained by subjective microscopic evaluation, we also performed quantitative analysis of the fluorescent intensities (Fig. 3) comparing sperm before the AR (panel A) and after the AR (panel B). The fluorescent intensity lines for CD46 and β1 integrin involving the acrosome cap, equatorial segment and the post-acrosome region clearly showed the redistribution of fluorescent signals from AC to ES and WSH including PAR after the AR induction. We also performed a statistical comparison of fluorescent intensity data (panel C). Here, the statistical output comparing intensities of sperm before and after AR was in the accordance with our subjective analysis showing statistically significant increase of the intensities in ES and PAR after AR with a higher difference in the fluorescent intensity related to β1 integrins compared to CD46 (not subjected to statistical comparison).

### CD46 – β1 integrin interaction

**Figure 4:**
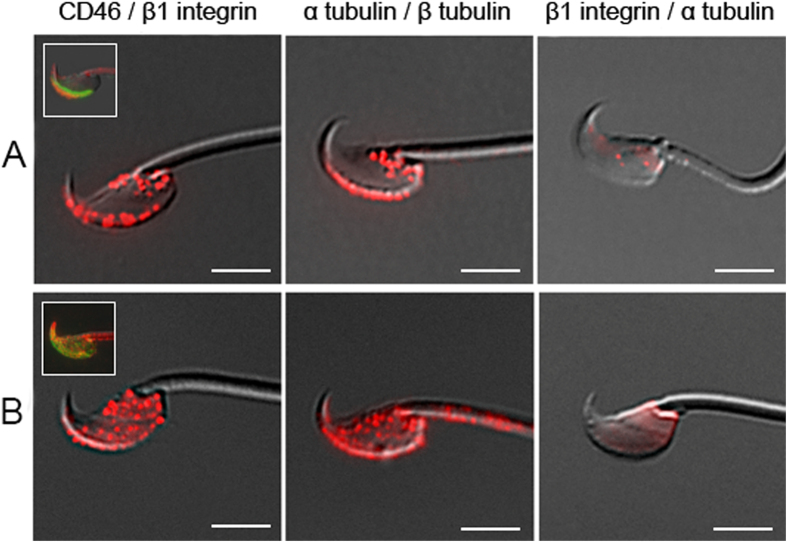
The study of protein-protein interactions. Interactions of CD46 and β1 integrin in C57BL/6 spermatozoa determined by Duolink proximity ligation assay. (**A**) freshly released sperm, (**B**) sperm during the induced AR. Positive control (α tubulin/ β tubulin), negative control (β1 integrin/ α tubulin). The small pictures in the corners represent the immunofluorescent dual staining of CD46 and β1 integrin, CD46 (green), β1 integrin (red). Scale bar represents 4 μm.

Based on a similar distribution of both the proteins, their parallel time relocation during the AR and published data from the somatic cells^17^, we decided to perform a specific proximity ligation assay that extends the limits of traditional immunofluorescence assays and enables direct detection of specific protein – protein interactions with a single molecule resolution. Besides the studied proteins (CD46 and β1 integrin subunit), the positive (α tubulin and β tubulin) and negative (β1 integrin and α tubulin) control protein pairs were selected. Using Proximity Ligation Assay Duolink (PLA) the proteins were adequately labelled (please, see methods for a detailed description) and evaluated under fluorescent microscopy (Fig. 4). Based on the obtained results, the CD46 - β1 integrin - protein interaction was proven in both freshly released sperm (Fig. 4A, first column) and sperm after the acrosome reaction (Fig. 4B, first column). The dynamic distribution of studied proteins over the sperm head was also visible by this assay and it is possible to compare it with standard fluorescent results in the small picture in the left hand corner of Fig. 4. A positive (Fig. 4A,B second column) and negative control (Fig. 4A,B third column) gave relevant positive/negative results. The assay was repeated twice with the same outcome.

### Stimulated emission depletion (STED) super–resolution microscopy

Dual immunofluorescent staining was used for the analysis of the accurate position of CD46 and integrins by STED super-resolution microscopy. The ability of STED microscopy to distinguish individual membrane structures of the mouse sperm head was previously published^39^. Thanks to a high lateral resolution of approximately 60 nm, this method enabled us to detect individual structures of mouse sperm head, such as plasma membrane, as well as the outer and inner acrosomal membrane, which helped us to investigate in detail the presence of studied proteins in each of them.

**Figure 5:**
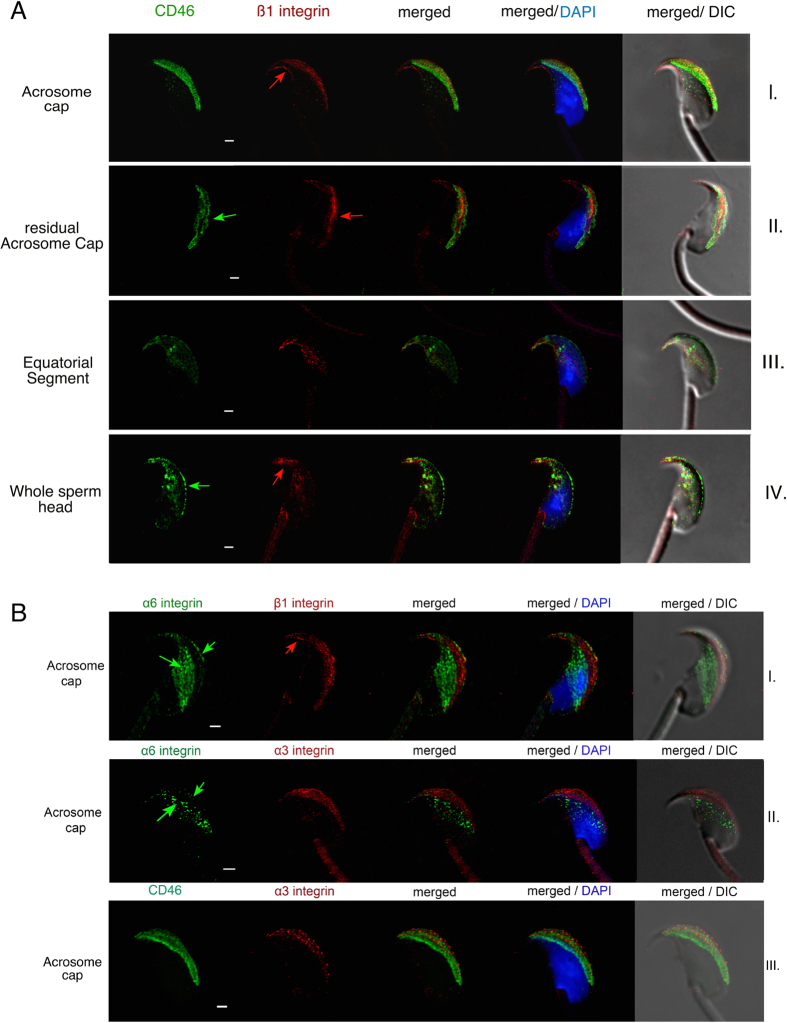
Dynamics of CD46 and β1/α6/α3 integrin captured by STED. (**A**) (line I) CD46 (green) is locked over the acrosome vesicle and β1 integrin (red) is confined to both the plasma and the outer acrosomal membrane with prominent labelling of the perforatorium (see the red arrow); (line II) the onset of AR with the membrane vesiculation is visible, as well as the visibility of CD46 and β1 integrin within the acrosome and plasma membranes (see green and red arrows); (line III) the loss of the acrosome including the CD46 and β1 integrin signal is visible. Relocation of CD46 and β1 integrin over the equatorial segment in patchy clusters can be recognised; (line IV) CD46 is localized through the inner acrosome membrane (see the green arrow), the equatorial segment and over the post-acrosome region. β1 integrin shows a similar pattern to CD46 and also remains localized in the perforatorium (see the red arrow). (**B**) (line I) α6 (green) and β1 (red) integrins occupy different regions of the intact sperm head, except the same localization in the plasma membrane over the acrosome and the hook. α6 integrin is continues to be spread over the equatorial segment, even prior to AR (see the green arrows). β1 integrin is further present in the outer acrosomal membrane (when compared with the CD46 dual staining in AI) and perforatorium (see the red arrows). (line II) The α3 (red) integrin pattern is clearly different to the α6 (green) subunit, but remarkably similar to the one of β1 (red, line I) in the acrosome region. α3 is expressed on in the outer acrosomal membrane and the plasma membrane over the acrosome. (line III) The differences in the localization of the α3 (red) integrin and CD46 (green) are visible. The α3 integrin subunit is detectable on the plasma (see also Fig. S3b for detail) and the outer acrosomal membrane, when CD46 is defined strictly to the acrosomal membranes only. DAPI (blue); Scale bar represents 1 μm.

**Figure 6:**
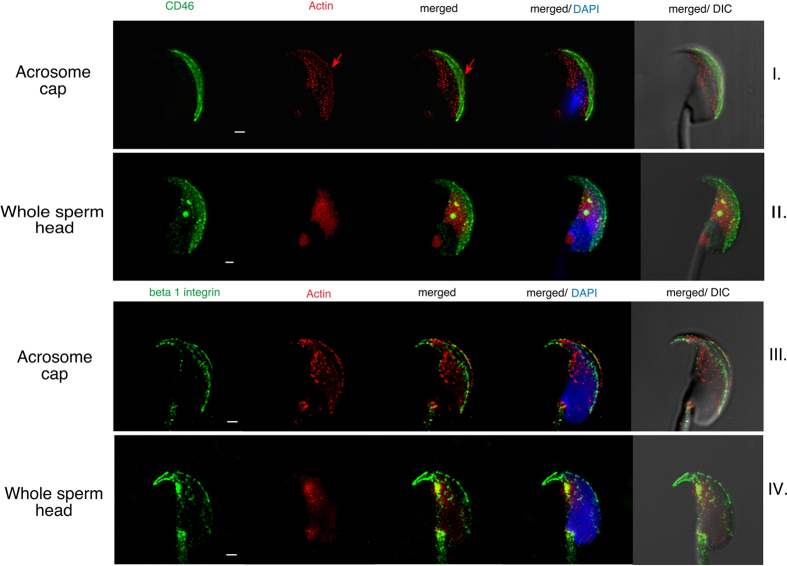
Dynamics of CD46, β1 integrin and actin captured by STED super-resolution microscopy. (line I and III) Actin (red) fills the apical acrosome and equatorial segment of an intact sperm head, it copies the plasma membrane and overlays the CD46 (green, line I) labelled acrosome in a thin line pattern (see red arrow). (line III) β1 integrin and actin display a similar localization in the apical acrosome cap region (plasma and acrosomal membrane). The perforatorium is filled with actin, but clearly marked with the β1 integrin. (line II and IV) Actin (red) is confined to the equatorial and postacrosomal segment in the acrosome reacted sperm. CD46 (green, line II) and β1 (green, line IV) relocation progress during the AR is visible. DAPI (blue); Scale bar represents 1 μm.

A detailed mutual localization of CD46 and β1 integrins was visualised on freshly released epididymal sperm with an intact acrosome (Fig. 5AI, S2 video image). In this case, the presence of CD46 was detected solely in the acrosomal cap in both the outer and inner acrosomal membranes, but not on the plasma membrane covering the acrosome region. Contrary to that, the β1 integrin subunit was present in the plasma membrane where it was detected in the area of acrosome cap (AC) and the hook marking the shape of the apical and dorsal sperm head (Fig. 5AI; 5BI and Fig. 6III, see the arrows). Unlike CD46, β1 integrins are present in an intact sperm head only in the outer acrosomal membrane, but not in the inner one (Fig. 5AI) and only later during the AR is the protein relocated over the inner acrosome membrane, equatorial segment and eventually the whole sperm head.

**Figure 7:**
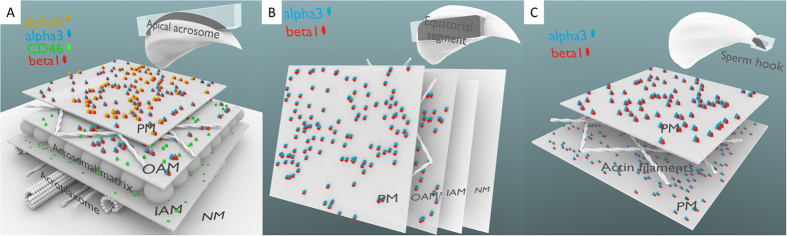
3D cartoon summarizing the localization of CD46 and α3, α6 and β1 integrins among different membrane structures of the intact sperm head. (**A**) Apical acrosomal area, (**B**) Equatorial segment, (**C**) Sperm hook. PM – plasma membrane, OAM – outer acrosomal membrane, IAM – inner acrosomal membrane, NM – nuclear membrane. Using our experimental techniques, we are not able to determine, if integrins α3 and β1 are present on both PM and OAM or exclusively on PM in the equatorial segment (panel B).

In order not to ignore α subunits of integrin proteins we decided to characterize the localization of α3 and α6 subunits, which were previously detected in sperm^25^,^40^. As shown in Fig. 5B, their localization was remarkably different. Based on these results, we can conclude that the α6β1 and/or α6β4 pair could be localized in the plasma membrane covering an intact apical sperm head including the hook. Due to an absence of β1 in the equatorial segment of intact sperm, it is probable that only the α6β4 pair would be localized in the equatorial region (Fig. 5BI, see the arrows), entirely excluding the acrosome vesicle. On the other hand, the α3β1 pair is confined to the plasma and outer acrosome membrane (Fig. 5BII,III, S3a–c left), however, the sperm hook is not labelled by α3 integrins (Fig. 5BII,III), which gives us a clear difference between the α3 and α6 subunit of β1 integrins. The co-staining of the α3 integrin subunit with CD46 supports this observation and confirms that the α3 integrin localizes into the outer, but not inner acrosome membrane in intact sperm. This is clearly visible in Fig. 5BIII, as the CD46 labels the entire acrosome vesicle and its signal outspreads the one given by the α3 integrin subunit (Fig. 5BIII, see the arrow). The Estrogen receptor β (ERβ) labelling, which is excusive to the plasma membrane was used to double confirm the expression of α3 integrin (Fig. S3C right). A positive colocalization of ERβ with α3 integrin and a negative one with CD46, supports the identified α3 integrin plasma membrane localization. All the results addressing localisation of various forms of integrins and CD46 among individual membrane structures in the sperm head are then graphically summarised (Fig. 7).

Dramatic membrane reorganization starts in the acrosome cap region after the initiation of the acrosome reaction^41^. However, the membrane changes have not been studied in the area of the rodent specific apical hook yet. Using the STED super-resolution microscopy, we focused on the hook part of the falciform sperm head, which plays an important role in sperm-sperm assembly^42^. Our results show that the α6, β1 integrin subunits (Fig. 5AI,BI and Fig. 6III), localized in the plasma membrane of the apical hook, remain in their localization during the following membrane rearrangements during the AR (Fig. 5AII). However, after the AR is completed and the acrosome vesicle is lost, even the CD46 protein relocates to the apical hook as well as into the equatorial segment (Fig. 5AIII,IV). So in the end, both CD46 and β1 integrin subunit fill the entire sperm head including the hook (Fig. 5AIV and Fig. 6I,IV) where their mutual position is visible.

Regarding the apical hook, we also show in intact sperm the presence of β1 integrin in a “bridge” like three point structure (Fig. S4, see also 3D structure in S2) connecting the very tip of the hook with its ventral part and supporting the apical end of the nucleus. The presence of CD46 was completely absent though. This structure was previously described as a hook rim^43^, but its protein content or function remains unclear.

The important part of acrosome reaction is the relocation process of proteins involved in sperm-egg binding and fusion. The key role of actin cytoskeleton has already been described during this process^8^, which supports our findings of possible actin cytoskeleton participation in the relocation process of the studied proteins (Fig. 6). In the presented STED micrographs, actin cortical cytoskeleton (Fig. 6I) is visibly changing its pattern and localization according to the status of the acrosome cap and corresponds with the described dynamics of CD46 and β1 integrin (Fig. 6II) during AR.

### Super–resolution 3D Structured Illumination Microscopy (SIM) and colocalization analysis

**Figure 8:**
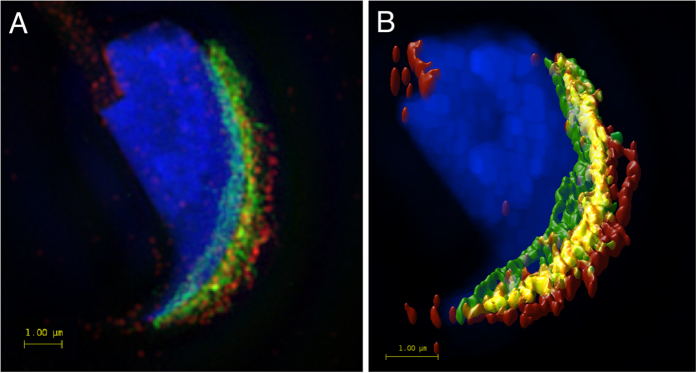
SIM super-resolution microscopy and visualization of mutual position of CD46 and β1 integrin. (**A**) SIM data show the localization of CD46 (green) on the inner and outer acrosomal membrane and β1 integrin (red) on the plasma and outer acrosomal and plasma membrane of the acrosomal area. Scale bar represents 1 μm. (**B**) SIM super-resolution image analysed by Huygens software, showing the colocalization area (yellow) of selected proteins in the outer acrosomal membrane. The colocalization map is based on Pearson’s correlation coefficient. Scale bar represents 1 μm. DAPI (blue).

Data collected by 3D super–resolution SIM was used for colocalization analysis of the mutual position of the CD46 and β1 integrin subunit. 3D-SIM was chosen to collect data for colocalization analysis due to its higher resolution in z-axis (in our case approximately 350 nm). The mutual position of the CD46 and β1 integrin subunit was visualised with dual immunofluorescence staining in freshly released epididymal sperm with an intact acrosome (Fig. 8A) and microscopy images were captured with a 3D SIM equipped microscope. Pearson’s correlation coefficient was used for the evaluation of the colocalization of the studied proteins^44^. Fluorescent images were analysed with Huygens software and the resulting values were statistically evaluated. The value of the average Pearson’s correlation coefficient of CD46 and β1 integrin was 0.784 ± 0.037. The result shows a high rate of colocalization of the studied proteins and it confirms the results obtained with the Duolink Proximity ligation assays (Fig. 4). The colocalization map by Huygens software, representing a visualization of Pearson coefficient, is shown in Fig. 8B and Fig. S9.

### CD46 and β1 integrin relocation affected by Latrunculin A

**Figure 9:**
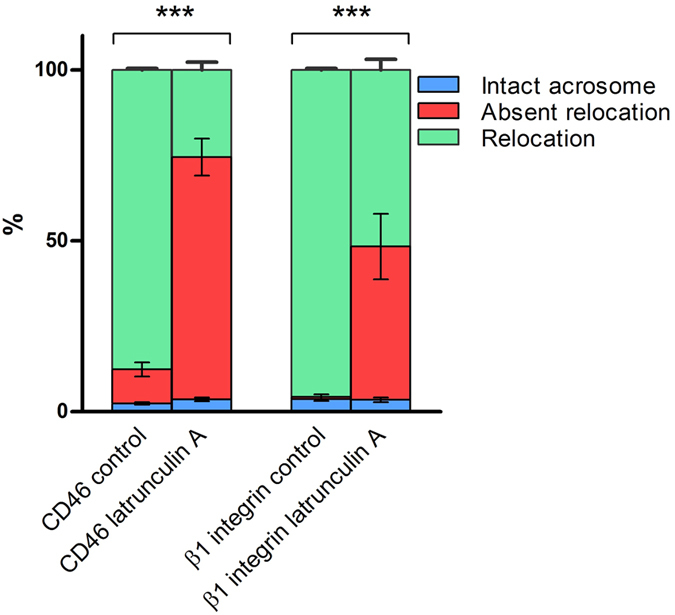
The differences in the percentage distribution of sperm with intact acrosome, sperm with absent relocation and sperm with protein relocation patterns of CD46 and β1 integrin after the AR induction between the control samples and samples incubated with Latrunculin A. Error bars represent standard deviations. ***p ≤ 0.001.

Latrunculin A, a toxin that binds to actin monomers and prevents them from polymerizing, significantly affected the ability of sperm to relocate both the CD46 and β1 integrin subunit when the acrosome reaction was induced (Fig. 9). The co-incubation of sperm with Latrunculin A followed by an induced AR led to a decrease of protein relocation of about 60% for CD46 and 40% for β1 integrin (Fig. 9). This compares to 90% positively relocated proteins in the control. There is also a significant difference in the percentages of sperm with a relocation pattern between CD46 and β1 integrin, where CD46 expresses a higher inhibition rate of the protein relocation compared to β1 integrin (significance indicators not shown in Fig. 9). Relevant control samples of sperm incubated with Latrunculin A and induced AR were run. The acrosome status was monitored by PNA lectin and there was near to zero blocking of the acrosome exocytosis caused by Latrunculin A (Fig. S5).

## Discussion

Protein relocation during the sperm acrosome reaction plays a crucial role for the ability of sperm to fuse with the egg as shown for the primary fusion protein IZUMO1^45^. Such a process is also true for spontaneous AR, in selected rodents, where IZUMO1 relocation speed correlates with the species-specific level of promiscuity^9^, stressing the importance of protein dynamics in the classical environment of sperm competition. Similarly, CD46 has been shown to play an important role in fertilization and in maintaining the acrosome integrity^13^,^14^,^15^. The mechanism by which the presence of CD46 in the acrosomal membrane stabilizes this organelle has so far remained unknown. In this study, we present proteins CD46 and β1 integrin subunit as a binding pair with their subsequent associations with the actin cytoskeleton. We bring evidence of the interaction between CD46 and β1 integrin, demonstrated by Proximity ligation assay and STED/SIM super-resolution microscopy. We present a dynamic relocation of CD46 and β1 integrins into the sperm fusogenic domain during the AR, and discuss their involvement in sperm-egg fusion. Moreover, we present that integrins α3 and α6 subunits are possibly pairing with β1, but occupying different compartments of the intact sperm head.

CD46 is a key membrane regulator of complement activation, which protects mammalian host cells from complement-mediated damage. Beside this role, CD46 is expressed in sperm solely as an unusual lower Mr hypoglycosylated isoform localized to the acrosomal cap and it only becomes surface exposed on the inner acrosomal membrane after sperm had acrosome-reacted^46^. Its role in fertilization was already noticed by using monoclonal antibodies to the first short consensus repeat (SCR1) ectodomain of CD46 which resulted in blocking the complement-independent interaction of human sperm with zona-free oocytes *in vitro*^47^,^48^. The recent discovery of a CD46 new physiological ligand Jagged-1^49^, which is highly expressed by the oocytes^50^ calls for readdressing the role of CD46 as a mediator of the initial steps of the sperm interaction with oolema. Implying the above and the fact that the acrosome reaction results in a morphological change and remodelling of the anterior sperm head, including the onset of protein network remodelling as a preparation for the gamete fusion, the presented relocation of CD46 during the AR further supports its possible interaction with an egg receptor, such as Jagged-1, inducing key signalling events for the initial steps of membrane fusions^51^. Delivering the evidence that CD46 was newly detected in the equatorial segment, by which mammalian sperm first touches the egg, would explain a previously made connection between CD46 reduced expression and an idiopathic infertility in humans^52^. Furthermore, we show that during later stages of the AR, when the acrosome vesicle is no longer present, CD46 location spreads over the whole sperm head (Figs 1A and 5A). This also suggests CD46 involvement in the later stages of sperm-egg fusion when sperm is being fully surrounded by the oolema and integrated within the ooplasma. The sperm specific CD46 isoform carries the cytoplasmic end 2^46^, which is responsible for signal transduction to the cytosol and IL-10 production in T- lymphocytes^53^. Giving a closer understanding of the CD46 sperm dynamics, its involvement in intracellular sperm signalling is very likely, however, its solitary behaviour is highly improbable. Despite the on-going research on CD46 in sperm, there has so far been no information on its binding partners or its suspected interaction with the cytoskeleton.

Here we demonstrated that one of the binding partners of CD46 on sperm is the β1 integrin subunit, which share the same acrosomal membrane localization and relocation behaviour. In intact sperm, we also identified, thanks to STED and SIM super-resolution microscopy, the localization of each protein across the individual apical head membranes and showed that the key integrin pair interacting with CD46 at the beginning of AR is α3β1 and not α6β1, which however could later play an important role in CD46’s protein organization. A previous interaction of CD46 and β1 integrins was detected in different somatic cell types^22^,^23^,^24^, as well as the presence of specific types of integrins in sperm^25^,^54^. Although the role of integrins in the fertilization process has been studied for a long time, it is mostly described in terms of the oocyte than in sperm. Interestingly, β1 integrin shows a patchy pattern nature in germ cells, as it was previously described both on the ooloema during oocyte maturation^55^ and in sperm^25^. Following our STED micrographs, the patchy localization is increasingly visible during relocation, especially into the ES (Fig. 5III) and may be relevant to the lipid raft dynamics. This is in correlation with previously shown α6β1 integrin clustering at the surface of the sperm head, which suggests its activation^25^. The fusing sperm could be expected to present this pattern, especially in the fusogenic region of the equatorial segment, and as this seems to be true for both CD46 and β1 integrins it just supports our findings of their interaction, and therefore a possible joint movement. Integrins as transmembrane receptors mediate not only static binding, but also dynamic adhesion processes between cells^40^. They are capable of transducing a signal in both directions, inside and outside of the cell^26^. Due to the integrin characteristics and the ability to bind to the actin cytoskeleton^56^, it was interesting to look into integrin distribution across the sperm head when actin was blocked. Giving the integrin nature, it did not come as a big surprise that the dynamic pattern changes considerably, which strongly supports the idea of integrin association with actin filaments in sperm, even though not necessarily all the integrins may possess the dynamic relocation nature, which may also differ based on the membrane location. It is puzzling so far whether the parallel change in CD46 dynamics (Fig. 9) is due to integrins or whether there can be an integrin independent CD46-actin binding in sperm. The report of an induction of a calcium flux on CD46 signalling^57^ may be particularly relevant to the involvement of CD46 in maintaining acrosome integrity and playing an active part in the onset of the acrosome reaction. This process is known to involve actin reorganization following protein kinase signalling and could be triggered, like in somatic cell, directly upon CD46 stimulation^59^,21 or by specific CD46 binding to β1 integrins and subsequent indirect associations through the integrin tail anchor to the surrounding cortical cytoskeleton^22^,^23^,^24^.

To present, 18α and 6β integrin subunits and 24 of their heterodimers have been described in mammals^58^,^59^. β1 integrin is one part of 12 and so it is the most represented integrin subunit of all^58^. Trying to concretely characterize β1 heterodimers interacting with CD46, we described the localization of α3 and α6 subunits that is known to form a heterodimer with β1, which was previously detected on sperm^25^,^40^. Up to now it has been described that the α3 subunit forms only part of the α3β1 heterodimer instead of the α6 subunit, which forms two heterodimers α6β1 and α6β4^58^. β4 was also detected on sperm where it forms a heterodimer with only α6^40^. We demonstrate the same localization of the α3 and β1 subunit on the plasma membrane surrounding the acrosomal cap and on the outer acrosomal membrane of acrosomal intact sperm probably forming the α3β1 heterodimer. In relation to its localization, it is probable that α3β1 is re-localized during the acrosome reaction and so it could play a role in sperm – egg binding. It is supported by the fact that in somatic cells, α3β1 associates with CD151 and CD81^60^. These proteins are also present on the egg^35^. Moreover, it was suggested that the α3β1 integrin is necessary for neuronal-glial recognition^61^. Thus α3β1 might participate in gamete recognition too. In the case of α6, we detected the same localization for α6 and β1 integrins in the plasma membrane of the acrosomal cap and the apical hook. At the same time we detected a localization exclusive for α6 without the presence of β1 in the plasma membrane of the equatorial segment. Thus it seems very probable that both α6β1 and α6β4 are present on mouse sperm. It was published^62^ that in the epithelial cell, α6β4, activates the signalling molecules of IP3 kinase and Rho kinase and affects the actin cytoskeleton, which can lead to the stimulation of other integrins such as α3β1. In sperm, IP3 kinase and Rho kinase are key signalling molecules that participate in the control of the acrosome reaction and actin cytoskeleton remodelling^63^,^64^,^65^.

Colocalization of the α6 and β1 subunit in the plasma membrane of the acrosomal cap area and apical hook indicate the possible presence of the α6β1 heterodimer in these structures. Moreover, the presence of α6β1in mouse sperm has been previously described^25^. Identification of the α6β1 integrin pair localization at the very end of sperm hook of the mouse sperm head may be of further importance. An unusual rodent sperm-sperm assembly into fast swimming sperm trains was previously described^42^,^66^, but the molecular basis of the assembly has not been discovered so far, apart from identifying a presence of actin cytoskeleton in the hook. Using a STED resolution, we show that α6β1 integrins are in the plasma membrane of the hook and therefore they are overlaying the previously documented position of actin^42^. Therefore, it seems likely that α6β1 integrins could also be involved in the formation of sperm trains. Moreover, we show a β1 integrin presence (Fig. S2,4) in a three point “bridge” like structure in the apical tip of the sperm hook, described as the hook rim^43^. This “bridge” is supporting the tip of the nucleus, anchoring it to the very tip of the hook. Supposedly, it has a resemblance of a sperm tail^43^, but this has not been further characterised with regards to a protein composition. We would expect there to be a membrane-surrounded structure based on the specific presence of β1 integrin, but due to a complete absence of CD46, it is unlikely to be formed by acrosome vesicle membranes. It could be, together with actin, actively involved in sperm hook deployment and the attachment to the sperm head or tail of another sperm, however, it still remains to be clarified.

To visually represent our new findings about the CD46 and β1 integrin localization and put them into the context of the known sperm membrane transformation processes occurring during AR, we have drawn 3D models and visualizations combining our own data with the data from other relevant publications. The topological localization of CD46 and β1 integrin molecules among individual sperm membranes is depicted in Fig. S6. In the intact sperm cells, β1 integrin occupied both the cytoplasmic and outer acrosomal membrane, but it is not present in the inner acrosomal membrane. On the other hand, CD46 molecules occupied both acrosomal membranes, but they are not present in the cytoplasmic membrane. During the fusion of the cytoplasmic and outer acrosomal membrane, both CD46 and β1 integrin are present in the newly formed structures called hybrid vesicles^67^. After the release of these vesicles, the surface of the sperm in the acrosomal area is newly formed by the intra-acrosomal part of the retaining inner acrosomal membrane, with the intra-vesicular domains of CD46 molecules exposed to the outer environment.

In Fig. S7, the potential mechanisms enabling the relocation of CD46 and β1 integrin molecules are addressed. Contrary to the acrosomal cap area, there is no fusion of the outer acrosomal membrane and the plasma membrane in the equatorial segment of the sperm head. The fusion takes place only at the interface between the acrosomal cap area and the equatorial segment (here we suggest naming it the ‘hybrid area’). In this specific location on the sperm head, the proteins from the outer acrosomal membrane are supposed to be able to relocate to the plasma membrane of the hybrid area and then to more distal parts of the equatorial segment. Furthermore, the hybrid vesicles resulting from the fusion of plasma and the outer acrosomal membrane are supposed to be able to re-fuse with the intact plasma membrane of other segments of the sperm head and thus relocate the material from the outer acrosomal membrane to the plasma membrane (both relocation mechanisms were suggested previously for the equatorin protein^68^. Finally, to also graphically address the temporal aspect of the relocation process, we generated a 3D simulation of the AR (Fig. S8), where the formation of hybrid vesicles, their release, together with the acrosomal matrix and the relocation of the CD46 and β1 integrin, are visualized. The simulation of the differential relocation speed of CD46 and β1 integrin is based on quantitative data sets from the population of sperm presented in Figs 2 and 3. This simulation thus enables one to visually approximate the most likely scenario of the relocation process in the average sperm cell.

In conclusion, we have shown that: 1) CD46 relocates from the acrosome into the equatorial segment and over the whole sperm head during the acrosome reaction, which is an important protein behaviour prior to sperm-egg fusion; 2) CD46 associates with the β1 integrin subunit and shares the same relocation pattern, which is disrupted when actin cytoskeleton is blocked, stressing their involvement in specific signalling pathways; 3) α3β1, but not the α6β1or α6β4 integrin pair, is confined to the intact acrosome vesicle and is consequently responsible, together with CD46, for maintaining its stability; 4) α6β1 integrins are localized to the apical hook of the intact mouse sperm head, which plays a role in the sperm train assembly.

## Materials and Methods

### Animals

Inbred C57BL/6 mice were obtained from a breeding colony of the Laboratory of Reproduction, Faculty of Science, Charles University. Mice were housed in the animal facilities of the Faculty of Science, Charles University, and food and water were supplied ad libitum. The male mice used for all experiments were a reproductive age of 10–12 weeks. All animal procedures and all the experimental protocols were approved by Local Ethics Committee of Faculty of Science, Charles University, carried out in strict accordance with the Animal Scientific Procedure, Art 2010, and subjected to review by this Local Ethics Committee of the Faculty or Science, Charles University, Czech Republic (accreditation no. 247732008-10001).

### Capacitation

Sperm from the distal regions of cauda epididymis were released into a 200 μl droplet of M2-fertilising medium (Sigma Aldrich, M7167) under paraffin oil (P-Lab, Czech Republic, P14501) in a Petri dish and pre-tempered at 37 °C in the presence of 5% CO_2_. Released sperm were assessed for motility and viability under a light inverted microscope with a thermostatically controlled stage at 37 °C. Sperm stock was diluted to the required concentration (5 × 10^6^/ml) in 100 μl of M2 medium under paraffin oil. Sperm were left freely to capacitate. Sperm samples were collected at both 60 and 90 min experimental times or the incubation was continued by an induction of the acrosome reaction. The freshly released epididymal sperm, which had not undergone capacitation, were used for protein detection to monitor protein status prior to capacitation.

### Acrosome reaction induction

Spermatozoa from the distal regions of the cauda epididymis were capacitated as described above. AR was induced by Calcium Ionophore (A23187 (CaI), Sigma Aldrich) at a final concentration of 5 μM. At both experimental capacitating times of 60 and 90 min CaI was left in the M2 medium for 60 or 90 min. All the sperm samples were incubated at 37 °C under 5% CO_2_.

### Monitoring of sperm quality and acrosome status

All the sperm samples were incubated at 37 °C under 5% CO_2_. Sperm motility and viability were assessed at every experimental time point, when a drop of spermatozoa was placed onto a glass slide and 2.5 mM PNA lectin (Molecular Probes, L-32458) was added. The status of the acrosome was examined immediately under a fluorescent microscope.

### Immunofluorescent detection of CD46 and β1 integrin subunit

Sperm smears were prepared for every *in vitro* incubation time stated above. Sperm were washed twice in PBS, smeared onto glass slides and air-dried. Sperm smears were fixed with 3.7% formaldehyde in PBS (pH 7.34) at room temperature for 10 min, followed by washing in PBS. Sperm were blocked with 10% BSA in PBS for 1 h and incubated with: primary antibody anti β1 integrin (sc-8978, Santa Cruz Biotechnology, Inc) diluted 1:10 in PBS and/or primary antibody anti-CD46 MM10 (HM-1118, Hycult Biotech) diluted 1:50 in PBS over night at 4 °C, followed by Alexa Fluor 488 goat anti-rabbit IgG or Alexa Fluor 568 donkey anti-rabbit IgG (Molecular Probes, Prague, Czech Republic) and/or Alexa Fluor 488 donkey anti-rat IgG (Molecular Probes, Prague, Czech Republic) secondary antibodies 1:300 in PBS for 1 h at room temperature. In case of dual staining, both secondary antibodies were applied together. Furthermore, PNA lectin (Molecular Probes, L-32458) was added at a concentration of 2.5 mM in PBS. After washing, the slides were mounted into a Vectashield mounting medium with DAPI (Vector Lab., Burlingame, CA, USA). The samples were examined with an Olympus IX81 fluorescent microscope and photographed with Hamamatsu ORCA C4742-80-12AG, using Olympus Soft Imaging Solutions software (Laboratory Imaging Ltd., Prague, Czech Republic). Representative results are shown.

For every experiment, we collected sperm data from eight mice. The positive or negative signal was evaluated from a total of 200 spermatozoa on every slide. In each group, at least two samples were analysed. Data were analysed statistically.

### Super-resolution microscopy

Freshly released, capacitated and acrosome reacted sperm were used for STED and SIM super-resolution microscopy. Sperm were collected, as described previously, with the following differences. Sperm samples were always prepared onto high precision cover glasses (thickness No. 1,5 H, 170 μm ± 5 μm, Marienfeld). Moreover, after the application of the primary and secondary antibodies, sperm were incubated for 5 minutes with DAPI (0.85 μg/ml, Thermo Scientific) and washed 3x in PBS. At the end, sperm were washed 1x in distilled water and air-dried. Dry samples were covered with 90% glycerol with 5% anti-fade N-propyl gallate (Sigma Aldrich). In the case of anti-actin labelling the sperm were fixed for 10 minutes in 3.7% formaldehyde at room temperature, centrifuged and immediately incubated with NH_4_Cl v PBS for 15 minutes. After washing 3x in PBS, sperm were smeared onto a glass slide and air-dried. Fixed sperm were permeabilized with acetone for 7 minutes in −20 °C. Further steps were the same as those described previously. For STED and SIM visualization, the following antibodies were used: primary antibodies anti- β1 integrin (sc-8978, Santa Cruz Biotechnology, Inc) 1:10 in PBS, anti-CD46 MM10 (HM-1118, Hycult Biotech) 1:50, anti-actin clone Ac-40 (A4700, Sigma Aldrich) 1:100, anti-α3 integrin (N19) (sc-6588, Santa Cruz Biotechnology, Inc) 1:10, anti-α3 (H-43) (sc-28665 Santa Cruz Biotechnology, Inc) 1:10, anti α6 integrin (F6) (sc-374057, Santa Cruz Biotechnology, Inc) 1:10, anti-estrogen receptor β H-150 (sc-8974, Santa Cruz Biotechnology, Inc) 1:50; secondary antibodies Alexa Fluor 568 donkey anti-rabbit IgG, Alexa Fluor 488 donkey anti-rat IgG, Alexa Fluor 568 goat anti-mouse IgG, Alexa Fluor 488 goat anti-mouse IgG, Alexa Fluor donkey anti-goat 568 IgG (Molecular Probes, Prague, Czech Republic) 1:300 in PBS. Fluorescent images were collected with a Leica TCS SP8 STED 3X microscope and DeltaVision OMX™ with the Blaze SIM Module microscope (Microscopy Centre, IMG AS, Prague, Czech Republic). Huygens Professional version 16.05 (Scientific Volume Imaging, The Netherlands, http://svi.nl) software was used for deconvolution of STED images and 3D visualization. An open source software Fiji was used for other image processing.

### Monitoring of Latrunculin A effect on CD46 and β1 integrin relocation

Sperm were left freely to capacitate for 60 min with the subsequent induction of the acrosome reaction (described above). Latrunculin A at a final concentration of 10 μM was added into the capacitating medium at the beginning of the 60 min sperm capacitation. AR was induced by 5 μM CaI, and sperm were incubated for another 60 min. Control sperm samples were prepared in the same way but without the addition of Latrunculin A. To detect the possible influence of the ongoing acrosome reaction by Latrunculin A, control samples were prepared; sperm were incubated with Latrunculin A both during capacitation and induction of AR. The status of the acrosome was detected using PNA lectin.

### Protein – protein interactions

To detect the interaction of proteins CD46 and β1 integrin, Proximity Ligation Assay Duolink (PLA) was used. Proteins α tubulin (DM1A, Sigma, 1:20) and β tubulin (sc-9104, Santa Cruz Biotechnology, Inc, 1:10) were selected as a positive control (DUO92101 Duolink^®^ *In Situ* Red Starter Kit Mouse/Rabbit, Olink Bioscience), β1 integrin (sc-8978, Santa Cruz Biotechnology, Inc) and α tubulin (DM1A, Sigma) as a negative control (DUO92101 Duolink^®^ *In Situ* Red Starter Kit Mouse/Rabbit, Olink Bioscience). The interaction of experimental proteins CD46 and β1 integrin was studied using a specially prepared starter kit, which was (inter alia) comprised of one PLA probe (DUO92005 Duolink^®^ *In Situ* PLA® Probe Anti-Rabbit MINUS, Olink Bioscience) and one Probemarker kit (DUO92009 Duolink® *In Situ* Probemaker PLUS, Olink Bioscience) was used to prepare the PLA probe anti-rat, using an unconjugated secondary antibody (A18741 donkey anti rat IgG, Thermo Fisher Scientific) which was conjugated with a short DNA strand.

Freshly released sperm and sperm after CaI induced AR (see above) were washed twice in PBS, smeared onto glass slides and air-dried. Sperm smears were fixed with 3.7% formaldehyde in PBS (pH 7.34) at room temperature for 10 min, followed by washing in PBS. Sperm were blocked with 10% BSA in PBS for 1 h and incubated with primary antibodies. In each experiment, two primary antibodies were used, each directed against one of the target proteins. These antibodies were raised in different species. Species-specific secondary antibodies (PLA probes) bind to primary antibodies, and each of them has a unique short DNA strand attached to it. Both DNA strands interacted through a subsequent addition of two other circle-forming DNA oligonucleotides, forming a DNA circle, which was closed by DNA Ligation. DNA circles were amplified using a DNA polymerase. The amplified DNA was detected by hybridization with labelled oligonucleotides, which produced a visible fluorescent spot. These spots were detected with an Olympus IX81 fluorescent microscope and photographed with Hamamatsu ORCA C4742-80-12AG, using Olympus Soft Imaging Solutions software (Laboratory Imaging Ltd., Prague, Czech Republic). Representative results are shown.

### SDS–PAGE immunoblotting

SDS electrophoresis and immunoblotting technique were used for the β1, α3 and α6 integrin and CD46 protein detection was performed by protocols based on standard methods^69^,^70^. A suspension of noncapacitated sperm from a sperm stock released from cauda epididymis was used. The sperm solution was diluted with PBS and a sperm pellet was re-suspended in an equal volume of SDS–PAGE non-reduced sample buffer and heated at 97 °C for 3 min. Samples were run on 5% stacking and 10% running SDS–polyacrylamide gel using Precision Plus Protein Dual colour Standards (Bio-Rad) as MW markers. Proteins were then transferred onto a nitrocellulose membrane (BioRad,). Non-specific sites on the membrane were blocked with PBS-blocking solution for 1 h (5% skimmed milk and 0.05% Tween 20 in PBS). The nitrocellulose membranes were incubated with the primary antibody for 1.5 h, washed six times for 5 min with a wash solution (0.05% Tween-20 in PBS) and incubated with a peroxidase-conjugated secondary antibody for 1 h. Proteins were identified as follows; CD46: rat monoclonal antibody (MM10, Hycult Biotech), 1:100 followed by donkey anti rat IgG antibody conjugated to HRP (170 5046, Bio-Rad), 1:10000; β1 integrin: rabbit polyclonal antibody (M-106: sc-8978 Santa Cruz Biotechnology) 1:20, followed by a peroxidase goat anti rabbit IgG (170–6515 BioRad), 1:3000; α3 integrin: primary rabbit polyclonal antibody (H-43): sc-28665 Santa Cruz Biotechnology diluted 1:20 and peroxidase goat anti rabbit IgG secondary antibody conjugated to HRP (170–6515 BioRad), 1:3000; α6 integrin: mouse monoclonal antibody (F-6): sc-374057 Santa Cruz Biotechnology 1:20, followed by a goat anti mouse IgG conjugated to HRP (170–6516 BioRad) 1:5000. Protein staining was visualized by chemiluminescence (Super Signal West Dura Extended Duration Substrate, Thermo Fisher Scientific). These experiments were performed at least three times with similar results. Representative results are shown.

### Statistical analysis

Experimental data were visualized and analyzed using STATISTICA 6.0. (Statsoft, Prague, Czech Republic) and GraphPad Prism 5.04 (GraphPad Software Inc., La Jolla, CA, USA). The differences in the relative fluorescent intensities between the acrosome-intact and acrosome-reacted sperm among individual relocation segments in Fig. 3C were analyzed by One-way analysis of variance (ANOVA) and Bonferroni’s Multiple Comparison Test. The differences in the percentage distribution of sperm with intact acrosome and sperm with protein relocation patterns for CD46 and β1 integrin between the control samples and samples incubated with Latrunculin A in Fig. 9 were analysed by the Mann-Whitney test. p value equal or lower than 0.05 was considered to be significant, *p ≤ 0.05, **p ≤ 0.01, ***p ≤ 0.001. Huygens Professional version 16.05 (Scientific Volume Imaging, The Netherlands, http://svi.nl) was used for the colocalization analysis and its visualisation. A colocalization analyser computed a Pearson’s correlation coefficient and created a colocalization map. The Pearson’s correlation coefficient expresses the rate of correlation of colocalizing channels in a dual-colour image and gives a value between minus 1 to plus 1. In this case, 1 means an absolutely positive correlation, 0 means no correlation and -1 means a perfect anti-correlation. The value between 0.5 and 1 is interpreted as colocalization. Costes method was used for a background estimation.

## Additional Information

**How to cite this article**: Frolikova, M. *et al*. Characterization of CD46 and β1 integrin dynamics during sperm acrosome reaction. *Sci. Rep.* **6**, 33714; doi: 10.1038/srep33714 (2016).

## Supplementary Material

Supplementary Information

Supplementary Video S1

Supplementary Video S2

Supplementary Video S3
