# Supplementary material for: Characterization of CD46 and β1 integrin dynamics during sperm acrosome reaction
Source: Sci Rep. 2016 Sep 26;6:33714. doi: 10.1038/srep33714 (PMC5036054; doi:10.1038/srep33714)
Supplement: Supplementary Information [file srep33714-s1.pdf]

Characterization of CD46 and β1 integrin dynamics during sperm acrosome reaction.

Michaela Froliková[1,2], Natasa Sebkova[1,3], Lukas Ded[1], Katerina Dvorakova-Hortova[1,2]*

[1]Group of Reproductive Biology, Institute of Biotechnology CAS, v.v.i., BIOCEV, Prumyslova 595, 252 50, Vestec, Czech Republic
[2]Department of Zoology and [3]Cell Biology, Faculty of Science, Charles University in Prague, Vinicna 7, Prague 2, 128 44, Czech Republic

*Corresponding Author
Katerina Dvorakova-Hortova
tel: +420 325873799
email: kathortova@gmail.com

Supplementary Figures

S1)

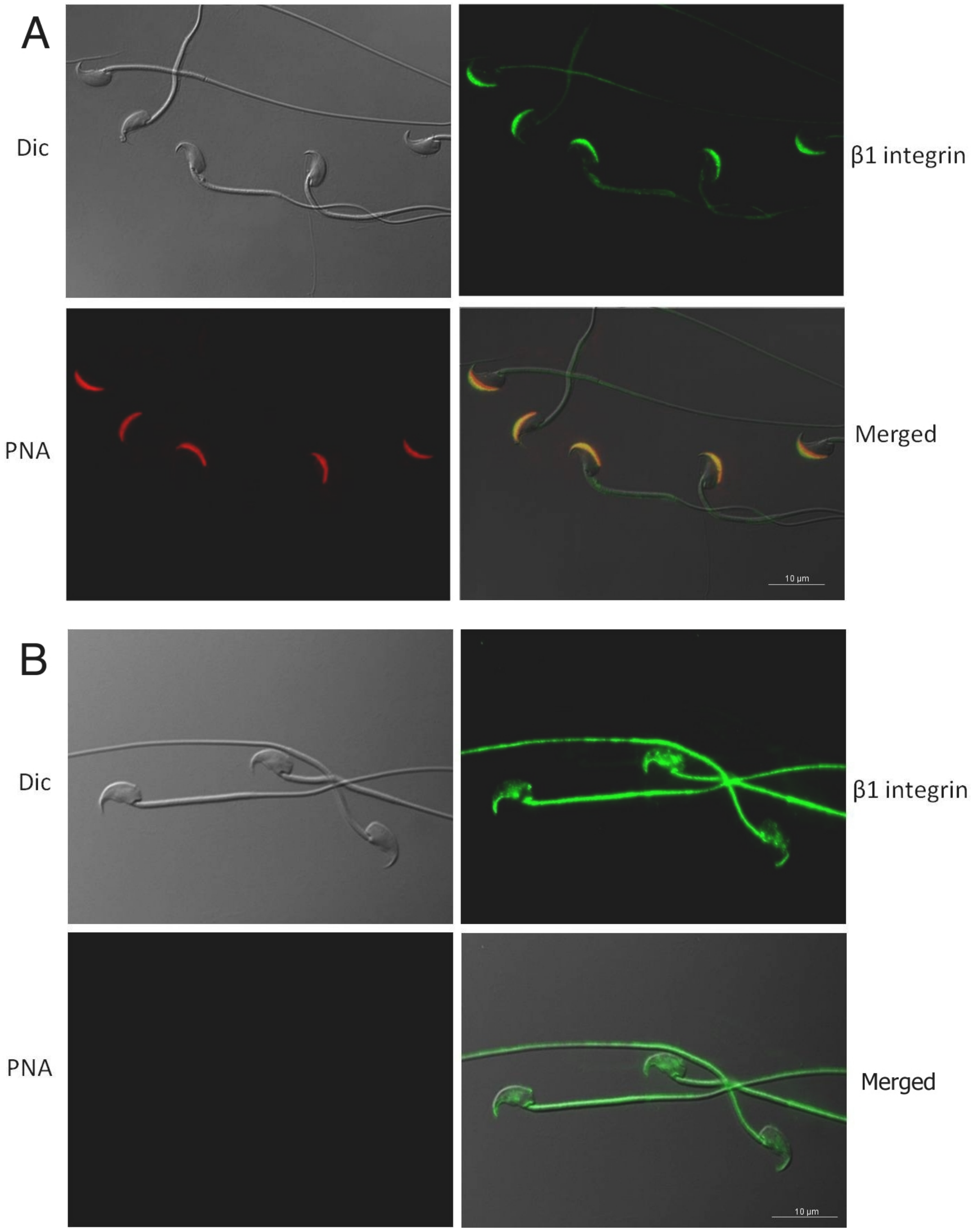

Figure S1: β1 integrin localization in mouse sperm head. (a) Acrosome intact sperm: β1 integrin (green) is localized in the apical acrosome cap labelled by PNA (red); **(b) Sperm after the acrosome reaction:** β1 integrin (green) covers the whole sperm head, PNA signal (red) is absent. Scale bar represents 10 µm.

S2)

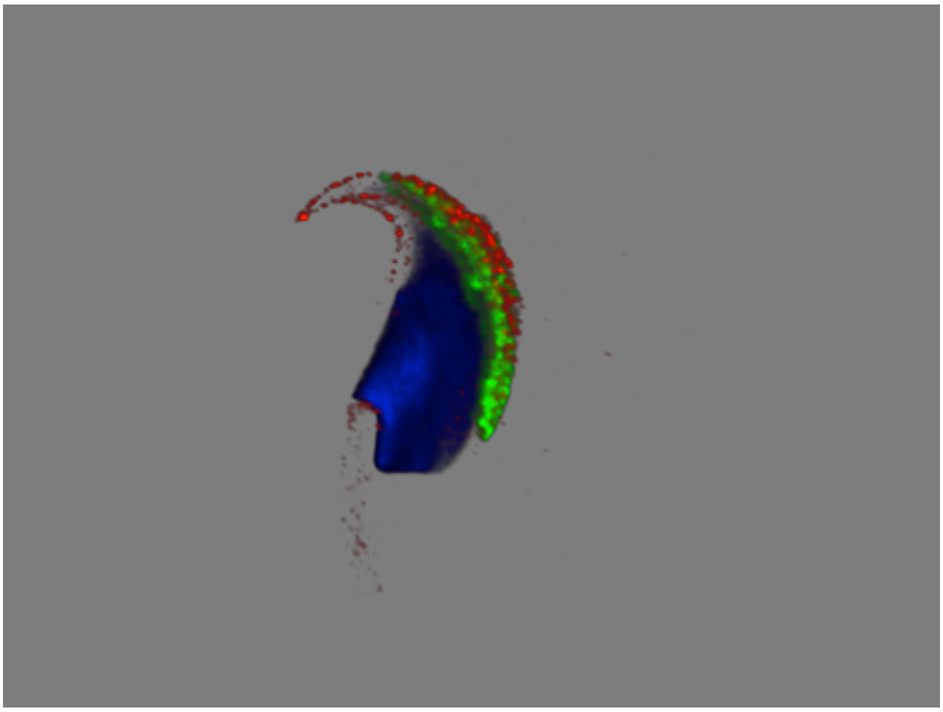

Figure S2: 3D visualization of structures in the apical hook and mutual position of CD46 and β1 integrin. CD46 (green) defines the acrosome vesicle; β1 integrin (red) overlays the apical dorsal part of the sperm head including the outer acrosome and plasma membrane. It also labels the “bridge” like structure (see also S4) of the hook rim; nucleus (blue). Please see the supplementary video.

S3)

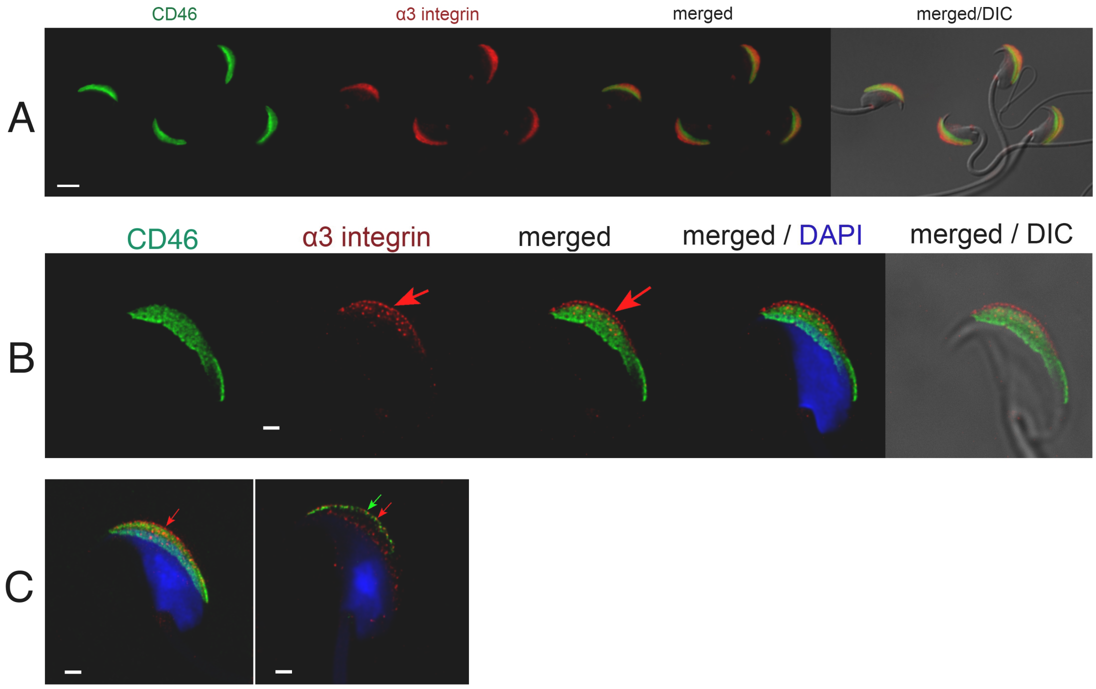

Figure S3: Mutual position of CD46 and α3 integrin or estrogen receptor β (ERβ). (a) epifluorescent microscopy; (b, c) STED super-resolution microscopy. (a, b, c left) CD46 (green) and α3 (red) integrin are expressed together on the outer acrosomal membrane of the acrosome intact sperm but only the α3 integrin subunit is present on the plasma membrane (see red arrows b, c left). **(c)** Dual staining of CD46 (green) - α3 (red) integrin subunit (left) and CD46 - ERβ (right) was used to support the α3 integrin localization. The expression of α3 integrin in the plasma membrane is clearly proven by negative colocalization with CD46, but positive colocalization with ERβ. Scale bar represents (a) 4 μm, (b, c) 1μm.

S4)

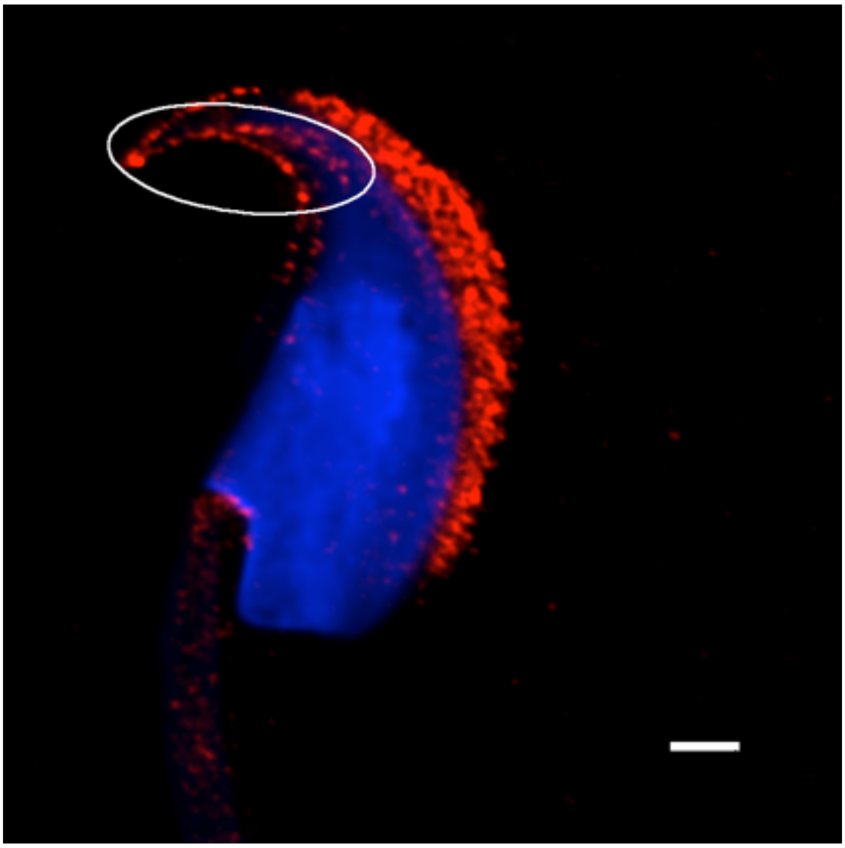

Figure S4: "Bridge" like structure in the apical hook labelled by β1 integrin.

β1 integrin (red) marks the three-point structure (in the white oval) (see also S2) underlying the tip of the nucleus and connecting it with the tip of the hook. The apical dorsal part of the sperm head including the outer acrosome and plasma membrane are also labelled; nucleus (blue). Scale bar represents 1μm.

S5)

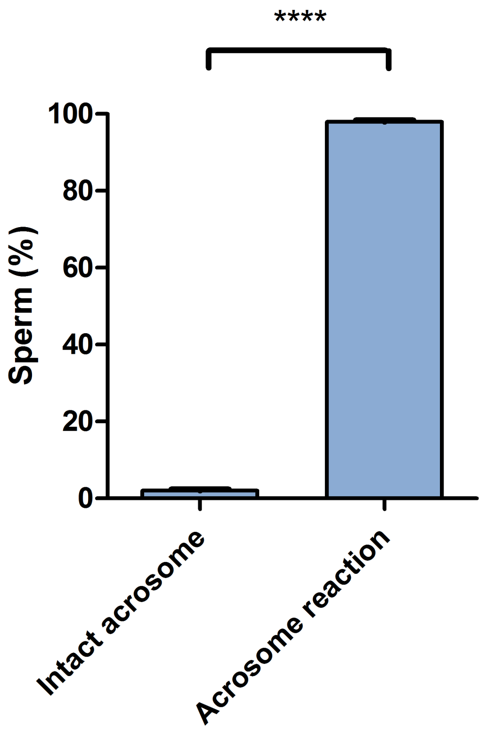

Figure S5: The differences in the percentage distribution of sperm with intact acrosome and acrosome reacted sperm. This graph shows the control sperm samples after the AR induction during incubation with Latrunculin A. The status of the acrosome was detected using PNA lectin. Error bars represent standard deviations. p value equal or lower than 0.05 was considered to be significant, $p \leq 0.001$***.

S6)

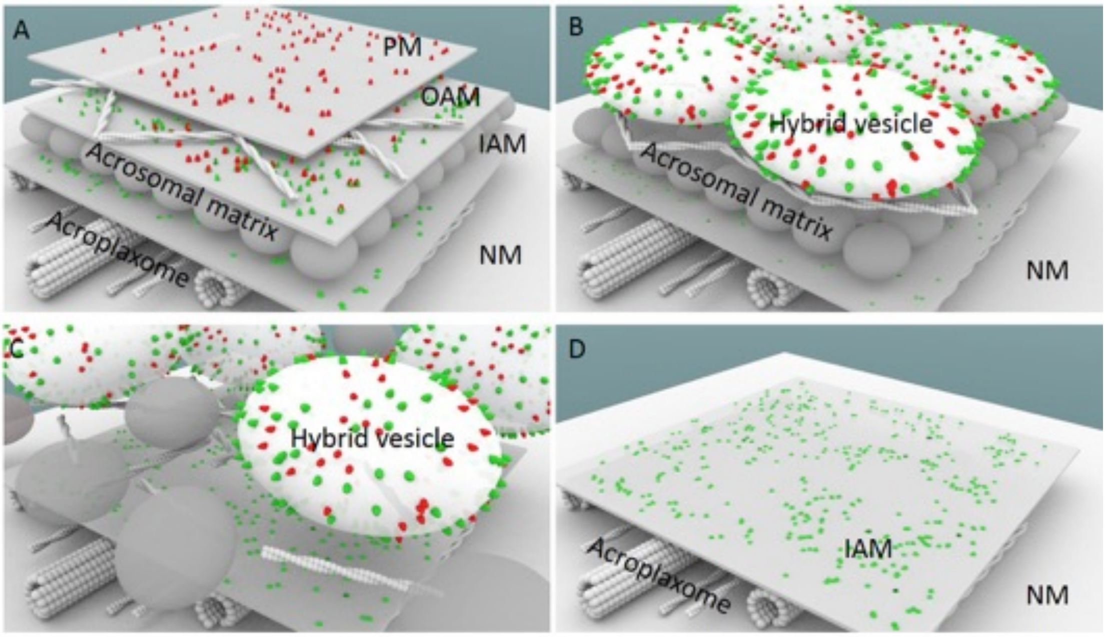

Figure S6: 3D visualisation of the distribution of CD46 (green) and β1 integrin (red) molecules among different membrane structures of the sperm head.

A – Intact sperm head in the acrosomal cap area. B – Fusion of the outer acrosomal membrane and plasma membrane resulting in the formation of hybrid vesicles containing material from both PM and OAM. C – Release of the hybrid vesicles and acrosomal matrix from the sperm head surface. D – Sperm surface in the acrosomal area is newly formed by the intra-acrosomal part of the retaining inner acrosomal membrane, with the intra-vesicular domains of CD46 molecules exposed to the outer environment. PM – plasma membrane, OAM – outer acrosomal membrane, IAM – inner acrosomal membrane, NM – nuclear membrane.

S7)

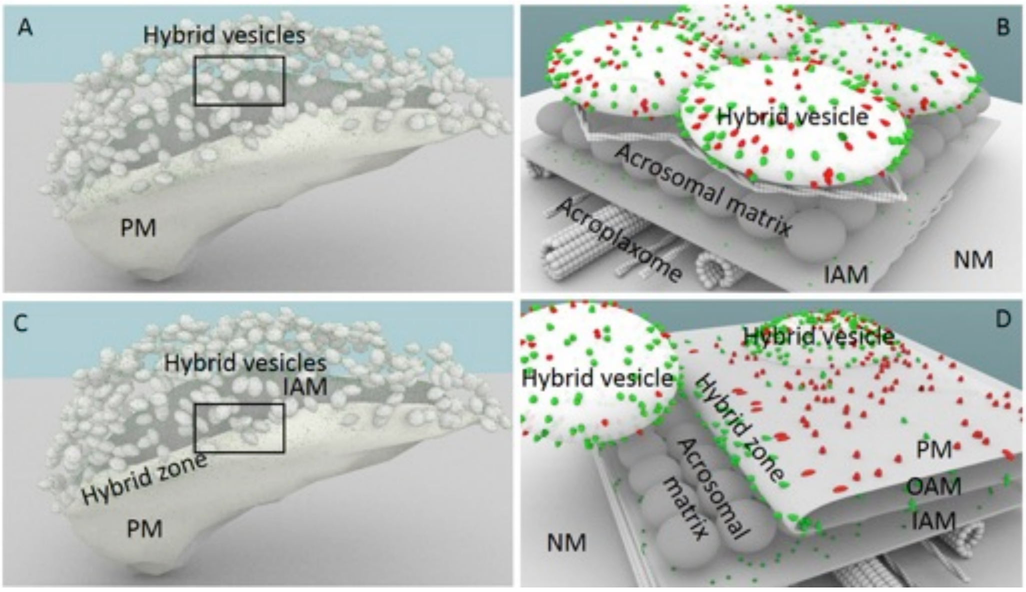

Figure S7: 3D visualisation of the potential membrane processes responsible for the relocation of the proteins from the acrosomal sperm area to the other compartments of the sperm head. A – The black rectangle indicates the hybrid vesicles resulting from the fusion of plasma and outer acrosomal membrane in the acrosomal cap area. B –Higher magnification of the area depicted in panel A showing 4 hybrid vesicles. These vesicles contain the membrane material from both PM (red) and OAM (green). C – The black rectangle indicates the interface between the acrosomal cap and equatorial segment areas of the sperm head (named here the hybrid zone). D – Higher magnification of the hybrid zone. In this zone, plasma and outer acrosomal membrane fusion results in the creation of the shelf-like membrane structure, where proteins from both membranes are suspected to be able to freely relocate to the other one. Furthermore, the hybrid vesicles are suspected to be able to re-fuse with the intact plasma membrane of other compartments of the sperm head and thus relocate the membrane material from PM and OAM and thus relocate the material to target areas. PM – plasma membrane, OAM – outer acrosomal membrane, IAM – inner acrosomal membrane, NM – nuclear membrane.

S8)

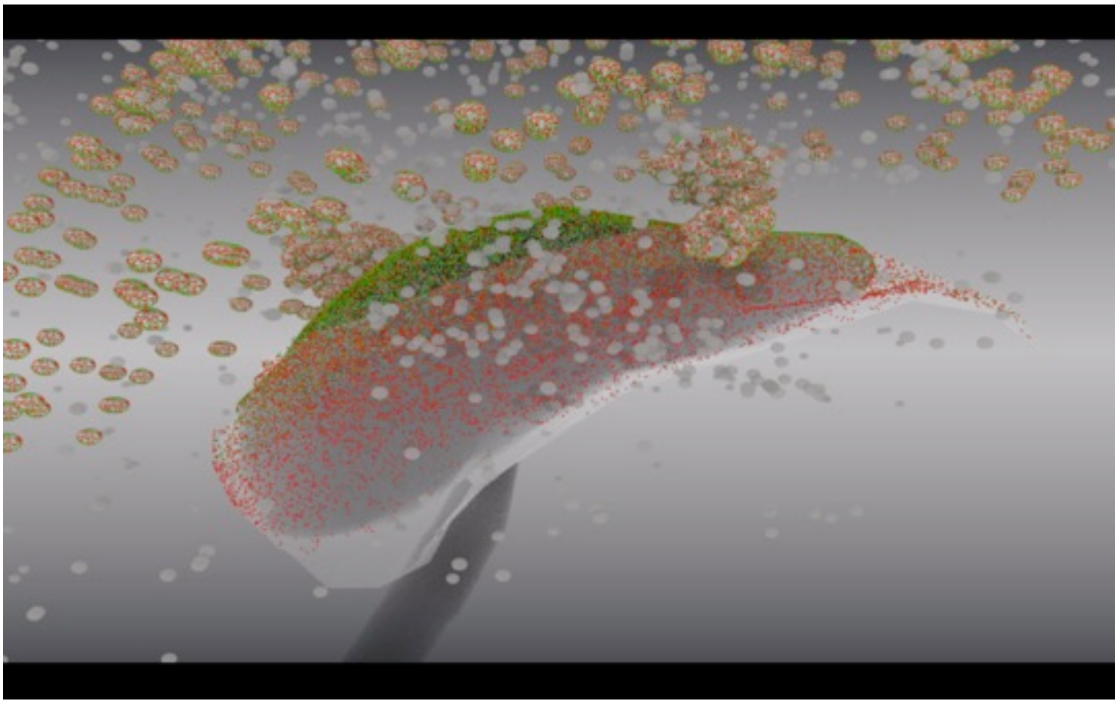

Figure S8: 3D visualization of the acrosome reaction showing the formation of the hybrid vesicles represented by lens-like structures with green (CD46) and red (β1 integrin) dots, which are released together with the acrosomal matrix content (represented by grey lens-like structures), followed by the relocation of CD46 and β1 integrin molecules to other compartments of the sperm head. The simulation of the differential relocation speed of CD46 and β1 integrin is based on the quantitative data sets from the population of sperm presented in figures 2 and 3 and the simulation visually approximates the most likely scenario of the relocation process in the average sperm cell. Please see the supplementary video.

S9)

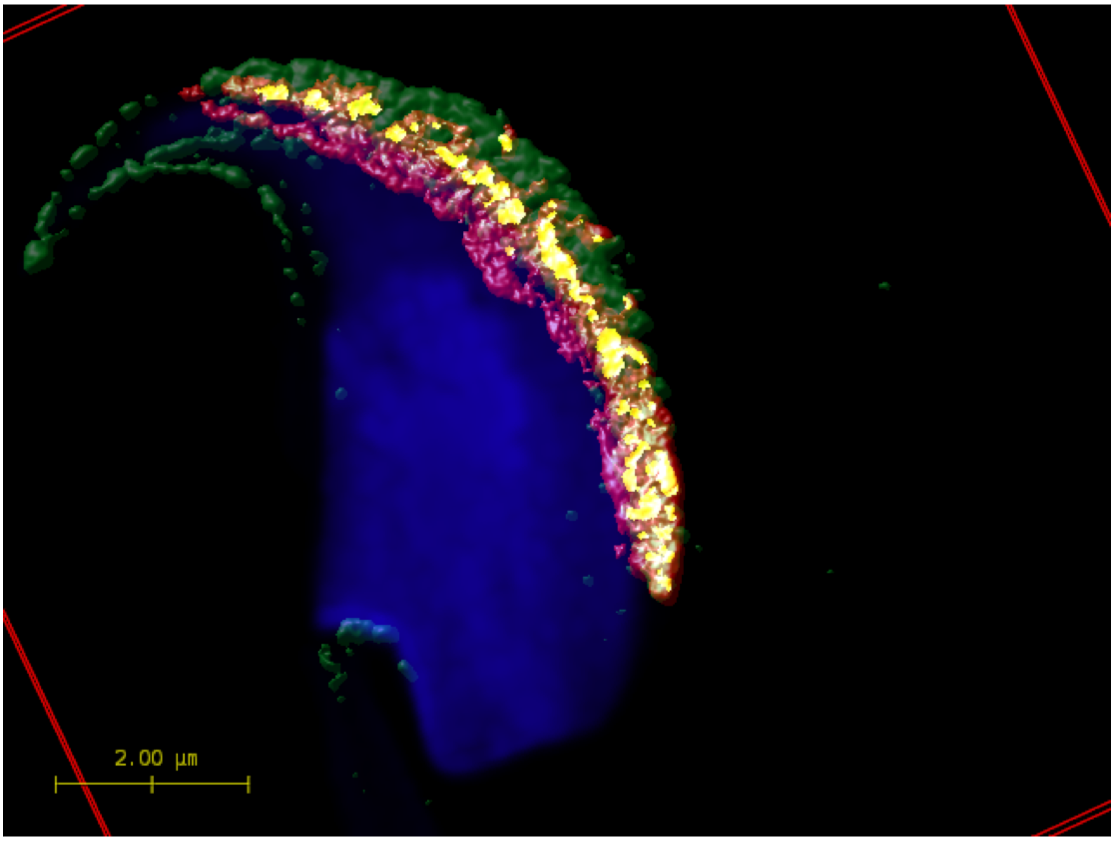

Figure S9: 3D visualization of CD46 and β1 integrin dual staining using Huygens software. CD46 (purple), β1 integrin (green), colocalization line (yellow), nucleus (blue). 3D visualisation offers a view of the mutual position of CD46 and β1 integrins in XYZ axes and allows a better resolution of the studied proteins and their localisation in individual membrane structures. The colocalization line is a visualization of Pearson's correlation coefficient and represents the place of the colocalization of both the studied proteins. This line correlates with the outer acrosomal membrane. Besides this mutual position, β1 integrins are detected in the plasma membrane of the acrosome cap and apical hook (green line on the sperm surface) and CD46 in the inner acrosomal membrane (inner purple line). Please see the supplementary video.

S10)

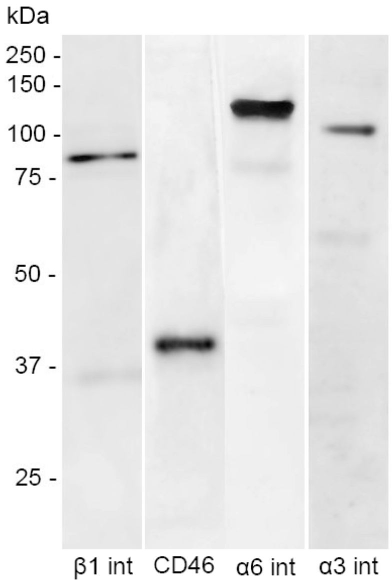

Figure S10: SDS–PAGE and western blotting immunoprotein detection in the whole sperm cell sample. β1 integrin corresponds to a band of molecular weight 89 kDa, α6 integrin 177 kDa, α3 integrin 125 kDa and CD46 44 kDa. Representative result shown.
